# Selective elimination of immunosuppressive T cells in patients with multiple myeloma

**DOI:** 10.1038/s41375-021-01172-x

**Published:** 2021-02-17

**Authors:** Mohamed H. S. Awwad, Abdelrahman Mahmoud, Heiko Bruns, Hakim Echchannaoui, Katharina Kriegsmann, Raphael Lutz, Marc S. Raab, Uta Bertsch, Markus Munder, Anna Jauch, Katja Weisel, Bettina Maier, Niels Weinhold, Hans Jürgen Salwender, Volker Eckstein, Mathias Hänel, Roland Fenk, Jan Dürig, Benedikt Brors, Axel Benner, Carsten Müller-Tidow, Hartmut Goldschmidt, Michael Hundemer

**Affiliations:** 1 Department of Hematology, Oncology and Rheumatology, Heidelberg University Hospital, Heidelberg, Germany; 2 Division of Applied Bioinformatics, German Cancer Research Center, Heidelberg, Germany; 3 Faculty of Biosciences, Heidelberg University, Heidelberg, Germany; 4 Department of Hematology and Oncology, Erlangen University Hospital, Erlangen, Germany; 5 Third Department of Medicine, University Cancer Center (UCT), University Medical Center (UMC) of the Johannes Gutenberg University, Erlangen, Germany; 6 German Cancer Consortium (Dktk), Partner Site Frankfurt/Mainz, Mainz, Germany; 7 Clinical Cooperation Unit Molecular Hematology/Oncology, German Cancer Research Center and Department of Internal Medicine V, University of Heidelberg, 69120 Heidelberg, Germany; 8 National Center for Tumor Diseases, Heidelberg University, Heidelberg, Germany; 9 Institute of Human Genetics, Heidelberg University Hospital, Heidelberg, Germany; 10 Department of Oncology, Hematology and BMT, University Medical Center of Hamburg-Eppendorf, Hamburg, Germany; 11 Asklepios Tumorzentrum Hamburg, AK Altona and AK St. Georg, Hamburg, Germany; 12 Department of Internal Medicine III, Klinikum Chemnitz, Chemnitz, Germany; 13 Department of Hematology, Oncology and Clinical Immunology, Düsseldorf University, Hamburg, Germany; 14 Department of Hematology, Essen University, Hamburg, Germany; 15 Division of Biostatistics, German Cancer Research Center (DKFZ), Heidelberg, Germany; 16 Molecular Medicine Partnership Unit, Heidelberg University Hospital, EMBL, Heidelberg, Germany

**Keywords:** Tumour immunology, Myeloma

## Abstract

Elimination of suppressive T cells may enable and enhance cancer immunotherapy. Here, we demonstrate that the cell membrane protein SLAMF7 was highly expressed on immunosuppressive CD8^+^CD28^-^CD57^+^ Tregs in multiple myeloma (MM). SLAMF7 expression associated with T cell exhaustion surface markers and exhaustion-related transcription factor signatures. T cells from patients with a high frequency of SLAMF7^+^CD8^+^ T cells exhibited decreased immunoreactivity towards the MART-1_aa26–35*A27L_ antigen. A monoclonal anti-SLAMF7 antibody (elotuzumab) specifically depleted SLAMF7^+^CD8^+^ T cells in vitro and in vivo via macrophage-mediated antibody-dependent cellular phagocytosis (ADCP). Anti-SLAMF7 treatment of MM patients depleted suppressive T cells in peripheral blood. These data highlight SLAMF7 as a marker for suppressive CD8^+^ Treg and suggest that anti-SLAMF7 antibodies can be used to boost anti-tumoral immune responses in cancer patients.

## Highlights

- SLAMF7 is a highly expressed marker on the surface of suppressive CD8^+^ T cells and its expression correlates with an exhausted phenotype in T cells.
- SLAMF7^+^ CD8^+^ Treg cells could be eliminated using anti-SLAMF7 antibody Elotuzumab via antibody-dependent cellular phagocytosis in vitro and in vivo.

## Introduction

Multiple myeloma (MM) is a plasma cell (PC) disease that leads to anemia, bone lesions, and eventually renal failure [1]. It is the second most common hematologic cancer, accounting for 10% of all hematological malignancies [2].

Signaling lymphocyte activation molecule family 7 (SLAMF7, also known as CS-1, CD319, and CRACC) is a receptor of the CD2 family that is overexpressed on myeloma cells, subsets of natural killer (NK) cells, macrophages, and T cells [3–5]. SLAMF7 is a self-adhesion receptor that exists in two isoforms, and it was originally identified as a coactivating receptor on NK cells [6]. While the biological significance of SLAMF7 on MM cells is not completely understood, some reports suggest that it may play a role in mediating self-adhesion to bone marrow (BM) stromal cells or contribute to MM cell growth and proliferation [7, 8].

The introduction of the SLAMF7-specific humanized antibody elotuzumab represented a milestone in MM treatment. Elotuzumab was one of the first approved antibodies for MM treatment, and it showed clinical efficacy in combination with lenalidomide or pomalidomide in patients with relapsed MM [9–13].

Recent data highlight the impact of elotuzumab on the immune system. SLAMF7 is expressed on NK cells, and increased antibody-dependent cellular cytotoxicity (ADCC) by NK cells after incubation with elotuzumab in vitro has been reported [14–16].

SLAMF7 is also expressed on several T cell subsets, but its functional role remained to be elucidated. PD-1 blockade increased the antitumor efficiency of elotuzumab in mice, a finding that hinted to a possible role of T cells in the mode of action [17]. SLAMF7-chimeric antigen receptor (CAR) T cells eliminate not only myeloma cells but also SLAMF7^high^ NK and T cells [18].

Signaling effects of SLAMF7 may differ between cell types. Ewing’s sarcoma’s/FLI1-activated transcript 2 (EAT-2) was shown to be essential for the signaling function of SLAMF7 after ligation in NK cells [15]. EAT-2 induced SLAMF7 phosphorylation, with subsequent activation of the downstream cascade [19]. Specifically, this activation in NK cells occurred only with the full-length isoform of SLAMF7 (SLAMF7-L) since the truncated isoform (SLAMF7-S) lacks the EAT-2 binding site in the immunoreceptor tyrosine-based switch motif. EAT-2 was expressed in NK cells but not myeloma cells. This finding might explain the different modes of action of elotuzumab in these two cell populations. Little is known about SLAMF7 and EAT-2 expression patterns and the signaling consequences of SLAMF7 in T cells, especially in those derived from MM patients. Based on the expression patterns of SLAMF7 and its signaling intermediates in various T cell subsets, elotuzumab may have distinct effects in various T cell subsets.

CD3^+^CD8^+^CD28^−^CD57^+^ T cells are a subset of CD8^+^ regulatory T cells (Tregs) that act as an immunosuppressive subset via soluble factors in a non-antigen-specific manner [20]. Interestingly, while CD8^+^CD28^−^CD57^+^ T cells isolated from the tumor microenvironment of cancer patients have immunosuppressive effects on cytotoxic T cells, they have no effect when isolated from the peripheral blood (PB) of healthy donors (HDs) [21]. This lack of immunosuppressive activity of CD8^+^CD28^−^ T cells isolated from HDs could be overcome by culture in the presence of interleukin (IL)-10 [20]. Recently, we showed that these Tregs were enriched in patients with MM and that their immunosuppressive capacity was induced by IL-10 [22]. Due to their relevance in patients with MM, we analyzed SLAMF7 expression on this phenotype during an induction therapy with lenalidomide, dexamethasone, and bortezomib with or without elotuzumab within the German Speaking Myeloma Multicenter Group (GMMG) HD6 clinical trial [23].

## Materials and methods

### Blood samples and ethics statement

To analyze SLAMF7 protein expression in T cells, PB/buffy coats from HDs (Institute for Immunology/IKTZ, Heidelberg University, Germany) and PB/BM from patients with MM were used. In accordance with the Declaration of Helsinki, all human studies were performed after obtaining written informed consent, and based on institutional guidelines, all human studies were approved by the Ethics Committee of the Medical Faculty at Heidelberg University. Data safety management was performed according to the data protection regulations of University Hospital Heidelberg.

### Molecular cytogenetic testing

Molecular cytogenetic testing was performed as previously described [24–26]. In brief, CD138^+^ BM PCs were purified using automated magnetic-activated cell sorting with anti-CD138 immunobeads as previously published [25]. For I Interphase fluorescence in situ hybridization (iFISH) analyses, a panel of two-color probe sets was used to detect numerical changes at the chromosomal loci 1q21/13q14, 5p15/5q35, 8p21/19q13, 9q34/15q22, and 11q22.3/17p13; the IgH translocations t(11;14)(q13;q32), t(4;14)(p16;q32), and t(14;16)(q32;q23); and any other IgH rearrangement. Hybridization was performed according to the manufacturer’s instructions (MetaSystems, Altlussheim, Germany), and a minimum of 100 interphase nuclei per probe were evaluated using an automated spot counting system (Applied Spectral Imaging, Edingen-Neckarhausen, Germany). Hybridization efficiency was validated using interphase nuclei obtained from HD BM, and the thresholds for gains, deletions, and translocations were set at 10%. The presence of deletion 17p and/or *t*(4;14) or *t*(14;16) was considered a high-risk cytogenetic profile [27].

### Cytokine profile screening for supernatants

After sample concentration, proteins were labeled at an adjusted concentration with scioDye 1 and scioDye 2 (Sciomics). The samples were incubated competitively using a dual-color approach on 10 scioCD antibody microarrays (Sciomics). Subsequently, slides were washed and dried.

Slide scanning was conducted using a Powerscanner (Tecan) with identical instrument laser power and constant PMT settings. Spot segmentation was performed with GenePix Pro 6.0 (Molecular Devices). Acquired raw data were analyzed using the linear models for microarray data (LIMMA) package of R-Bioconductor including normalization (specialized invariant Lowess method). For analysis of the samples a one-factorial linear model was fitted with LIMMA resulting in a two-sided *t*-test or *F*-test based on moderated statistics. The false discovery rate was controlled according to Benjamini and Hochberg.

### Preparation of human macrophages and measuring phagocytosis

Phagocytosis assay was performed with monocyte-derived macrophages as previously described [28]. Briefly, generated macrophages were co-incubated with isolated CPD (Cell Proliferation Dye eFluor® 670, Thermo Fisher) labeled T cells in the absence or presence of elotuzumab (10 µg/ml, E:T = 1:1, 37 °C) or an irrelevant IgG1 antibody (isotype control). Phagocytosis was monitored after 2 h by FACS or confocal microscopy (LSM700, Zeiss) at ×630 magnification.

### NSG mouse model

NOD.Cg-Prkdc^scid^IL2rg^tm1Wjl^/SzJ (NSG) mixed gender 8 weeks-old mice were injected s.c. with 2 × 10^6^ NCI-H929 myeloma cells in the right flank. T cells were isolated from PB of HD and were retrovirally transduced with a TCR for a novel HLA-A2.1-restricted myeloma associated antigen, that is the subject of a separate manuscript in preparation. 5 × 10^6^ TCR positive T cells highly expressing SLAMF7 were adoptively transferred (i.v.) 7 days later. All mice received an additional intraperitoneal (i.p.) injection of 7.2 × 10^5^ international unit (IU) human recombinant IL-2 on the day of T cell transfer to trigger T cell expansion. Mice were further divided into two groups that received either elotuzumab (200 µg per mouse, i.p.) or phosphate-buffered saline (PBS) on days 3, 10, and 14 after T cell transfer. Mice were sacrificed when the tumors reached 1 cm^3^ and TILs were isolated as described [29]. Briefly, freshly isolated tumor cells (from sacrificed animals) were dissociated by mincing the tissue with scalpels into 0.5-mm small pieces. Dissociated tissue was further triturated and filtered through a 100-mm cell strainer to obtain single-cell suspension. Cell suspension was then analyzed by flow cytometry to determine the frequency of the specific T cell populations. Animal experiments were performed according to approved protocol from the local animal welfare authorities of Rheinland-Pfalz (protocol AZ 23 177-07/G16-1-016).

### RNA sequencing

CD8^+^ T cells were isolated from MNCs of patients or HDs using a CD8^+^ T Cell Isolation Kit (Miltenyi Biotec, Bergisch Gladbach, Germany) according to the manufacturer’s protocol. The cells were then stained with antibodies and sorted using FACS according to a standard protocol into the SLAMF7^+^ and SLAMF7^−^CD8^+^ T cell populations. RNA was isolated using an RNeasy Mini Kit (Qiagen, Hilden, Germany) according to the manufacturer’s protocol, and RNA-Seq libraries were prepared using an Illumina RNA-Seq Preparation Kit and sequenced by a HiSeq 4000 with paired-end 100-bp sequencing, yielding 200 million reads per lane after passing quality control (QC) analyses in the Genomics and Proteomics Core Facility, German Cancer Research Center, Heidelberg, Germany. All raw data have been deposited at the European Genome-Phenome Archive (EGAS00001004915).

### Statistical and RNA sequencing analyses

The impact of elotuzumab on T cells expressing SLAMF7 was evaluated by analyzing paired patient samples before and after induction therapy by Wilcoxon’s signed rank test using the R computing environment (version 3.6.1).

Other comparisons between different patient groups were performed by *t*-tests using GraphPad (version 8.0) software.

A result was considered significant at *p* < 0.05 with ∗, ∗∗, and ∗∗∗ representing *p* < 0.05, *p* < 0.01, and *p* < 0.001 in graphical displays, respectively.

For RNA sequencing, RNA-paired FastQ files were aligned using STAR aligner (Version 2.5.3a) [30] to the reference genome (1KGRef_PhiX).

Sambamba (version 0.6.5) [31] was used to perform merging and duplicate marking of BAM files. Additionally, SAMtools software (version 1.6) [32] was used to perform a QC analysis using the SAMtools flagstat command and RNA-SeQC software (version 1.1.8) [33]. The featurecounts [34] implementation in the R/Bioconductor package subread (version 1.5.1) was used to perform gene-specific read counting over exon features based on the gencode 19 gene models. Both reads of a paired fragment were used for counting, and the quality threshold was set to 255. The gene expression data were then derived after preprocessing unnormalized read counts of all six samples (SLAMF7 positive and SLAMF7 negative) through several statistical learning methods in the R computing environment. Prefiltering was performed to include only nonzero reads per gene for the downstream analysis. The R/Bioconductor package DESeq2 (version 1.22.1) [35] was used to perform a differential expression analysis between SLAMF7-positive and SLAMF7-negative replicates by Wald tests within negative binomial generalized linear models. The procedure of Benjamini–Hochberg was then applied to calculate adjusted *p* values to control the false discovery rate at 0.05. To determine relevant effects, we used LFCs with a threshold of 2, and performed a shrinkage of effect size analysis (LFC estimates) using the R/Bioconductor package apeglm (version 1.4.2) [36] to approximate the posterior estimation for GLM to reduce variance for the genes with low information for statistical inference. DE Genes software was then used to perform a functional analysis (FA), and GSEA was performed using the R/Bioconductor package clusterProfiler (version 3.10.1) [37] and the MSigDB Collections database (C2: curated gene sets of canonical pathways) [38].

## Results

### Characterization of SLAMF7^+^CD8^+^ T cells by flow cytometry analyses

**Fig. 1:**
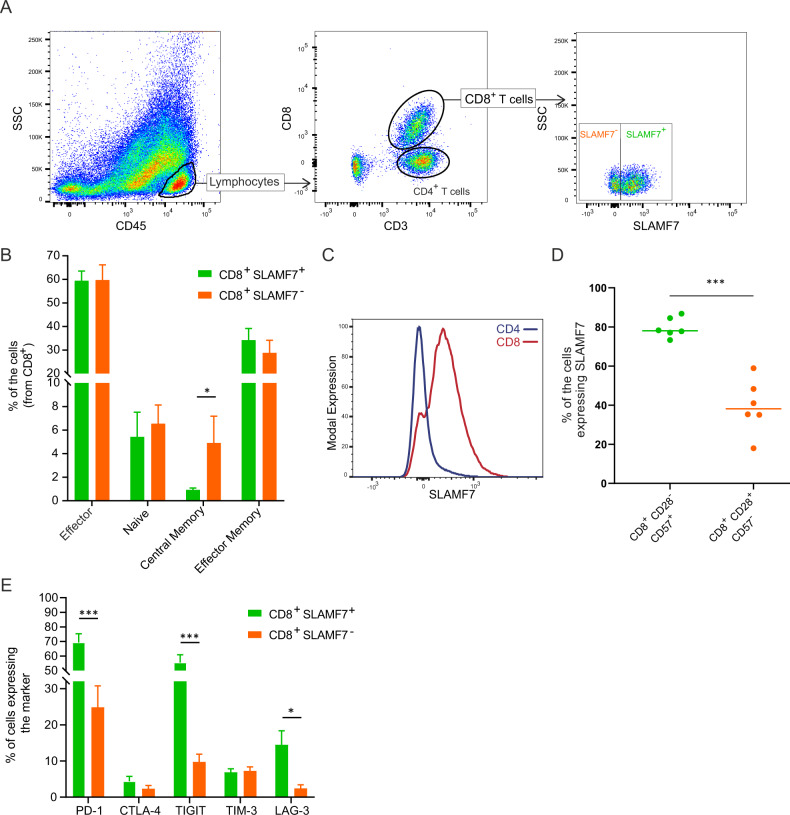
Characterization of SLAMF7^+^CD8^+^ T cells by flow cytometry analyses. **a** Representative gating strategy used to identify the percentage of SLAMF7-expressing CD8^+^ T cells (CD45^+^CD3^+^CD8^+^SLAMF7^+^) by flow cytometry. **b** The percentages of SLAMF7^+^ and SLAMF7^−^ T cells in each state (CD8^+^ effector: CD62L^–^CD45RA^+^; CD8^+^ naïve: CD62L^+^CD45RA^+^; CD8^+^ central memory: CD62L^+^CD45RA^−^; and CD8^+^ effector memory: CD62L^–^CD45RA^–^); T cells were analyzed in BM samples from NDMM patients (*n* = 9). **c** Representative histogram of modal SLAMF7 expression on the surface of CD8^+^ and CD4^+^ cells analyzed in BM samples from NDMM patients. **d** The percentages of SLAMF7-positive CD8^+^CD28^+^CD57^−^ (left) and CD8^+^CD28^−^CD57^+^ (right) cells from the BM of NDMM patients (*n* = 6). **e** The percentage of cells expressing exhaustion markers (PD-1, CTLA-4, TIGIT, TIM-3, and LAG-3) among SLAMF7^+^ and SLAMF7^-^CD8^+^ cells from the BM of NDMM patients (n = 4). Differences between groups were compared using either Student’s t-test or Wilcoxon matched-pairs signed rank test; ^∗^*p* < 0.05, ^∗∗^*p* < 0.01, and ^∗∗∗^*p* < 0.001.

BM samples from patients with newly diagnosed MM (NDMM) were analyzed for SLAMF7 expression by flow cytometry. SLAMF7 was substantially expressed on CD8^+^ T cells from MM patients (the percentage of CD8^+^ T cells expressing SLAMF7 varied between 1% and 92.7%, mean = 50.2%, Fig. 1a and Supplementary Fig. S1A). SLAMF7^+^ T cells in the central memory state were less frequent than SLAMF7^−^ T cells (Fig. 1b). SLAMF7 expression was very low on CD4^+^ T cells compared with that on CD8^+^ T cells (Fig. 1c). Furthermore, the frequency of SLAMF7-expressing CD8^+^ T cells was not different between the BM and PB compartments of NDMM patients (Supplementary Fig. S1B).

SLAMF7 was highly expressed on CD8^+^ Tregs, a subset characterized by the immunophenotype CD8^+^CD28^-^CD57^+^ (*p* < 0.0001, Fig. 1d). These CD8^+^ Tregs exert an immunosuppressive effect via soluble factors, and their occurrence directly coincides with the suppression of antigen-specific T cell responses in patients with PC dyscrasia [20, 22]. Of note, several tumor-associated exhaustion markers were expressed on a larger proportion of SLAMF7^+^CD8^+^ T cells compared to SLAMF7^−^CD8^+^ T cells. These exhaustion markers included TIGIT, a recently described protein that plays a role in the immune escape in MM [39–41], PD-1, and LAG-3 but neither CTLA-4 nor TIM-3 (*p* = 0.0002, *p* = 0.0004, *p* = 0.0229, *p* = 0.25, and *p* = 0.8, respectively, Fig. 1e). Overall, these findings suggested that SLAMF7^+^CD8^+^ T cells are in an exhaustion state.

Functionally, SLAMF7 knockout by CRISPR-Cas9 did not affect CD8^+^ T cell maturation and exhaustion (Supplementary Fig. S1C–E). Furthermore, secretion of vital cytokines such as IL-2, INFγ, GranzymB and suppressive cytokine IL-10 was not altered upon SLAMF7 knockout (Supplementary Fig. S1F). Accordingly, SLAMF7 was unlikely to initiate CD8^+^ T cell exhaustion pathways.

### RNA sequencing analysis of SLAMF7^+^CD8^+^ T cells

**Fig. 2:**
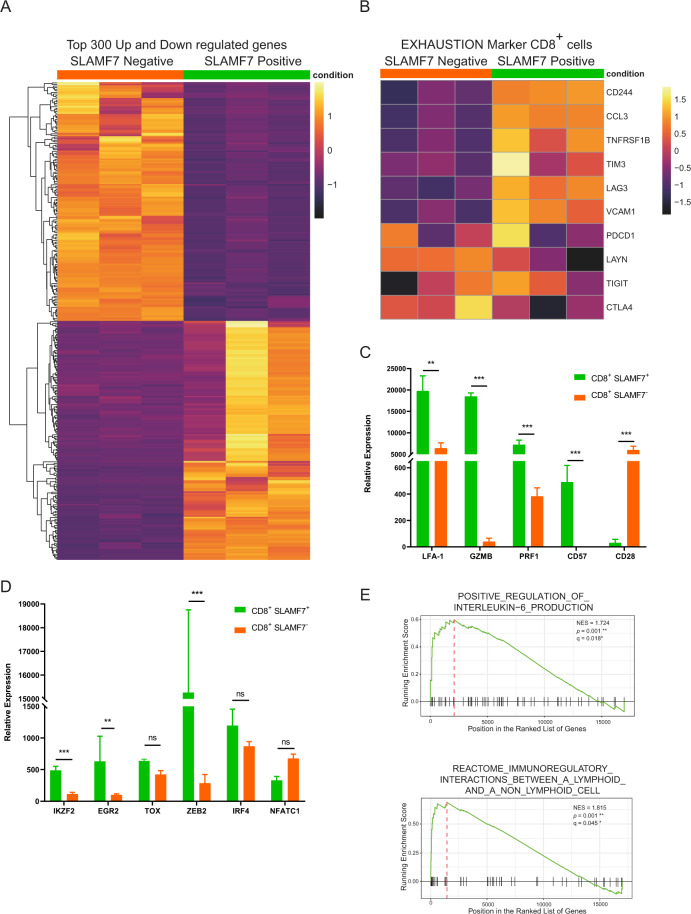
RNA sequencing analysis of SLAMF7^+^CD8^+^ T cells. **a** Heatmap showing the differential expression of the top 300 significantly up- and downregulated genes (LFC > 2, adjusted *p* value < 0.05) in SLAMF7^+^/SLAMF7^−^ CD8^+^ T cells (*n* = 3). A full list of all DE genes in SLAMF7^+^/SLAMF7^−^ T cells is shown in Data Table 1. **b** Heatmap showing the differential expression of exhaustion markers that were significantly elevated in CD8^+^ SLAMF7^+^ T cells compared with those in CD8^+^ SLAMF7^−^ T cells and exhaustion markers that were not consistently elevated (*n* = 3). **c** Relative expression of CD8^+^ Treg markers in SLAMF7^+^ and SLAMF7^−^CD8^+^ T cells (*n* = 3). **d** Relative expression of exhaustion-related transcripts in SLAMF7^+^ and SLAMF7^−^CD8^+^ T cells (*n* = 3). **e** GSEA plots showing the enrichment of “positive regulation of interleukin-6 production” and “reactome immunoregulatory interactions between a lymphoid and a nonlymphoid cell” in SLAMF7^+^CD8^+^ T cells (*n* = 3). Differences in gene expression levels were tested by Wald tests within negative binomial generalized linear models. The procedure of Benjamini and Hochberg (BH) was then applied to calculate the adjusted *p* values to control the false discovery rate at 0.05. To determine relevant effects, we used LFCs with a threshold of two.

We then performed an RNA sequencing of CD8^+^ T cells. CD8^+^ T cells from the PB of three patients with NDMM were sorted using fluorescence-activated cell sorting (FACS) into SLAMF7^+^ and SLAMF7^−^ groups, and RNA libraries were sequenced. Differential gene expression analyses identified 1662 genes that were significantly up- or downregulated (log_2_ fold-change [LFC] > 2, *p* value < 0.05) in SLAMF7^+^CD8^+^ T cells (Fig. 2a: top 300 up- and downregulated genes; a full list of differentially expressed genes in SLAMF7^+^/SLAMF7^−^ T cells is shown in Data Table S1). SLAMF7^+^CD8^+^ T cells expressed higher RNA levels of exhaustion markers, including LAG3, TNFRSF1B, CD244 (2B4), and TIM-3, than did SLAMF7^−^CD8^+^ T cells. PD-1 (PDCD1) and TIGIT RNA levels were not consistently upregulated in all samples, which differed from flow cytometry analyses of protein levels measured in BM samples that showed significant expression of PD-1 and TIGIT in SLAMF7^+^CD8^+^ T cells (Fig. 2b). Consistent with the flow cytometry analyses, SLAMF7^+^ T cells exhibited a phenotypic signature similar to CD8^+^ Tregs, characterized by the expression of LFA-1, GZMB, CD57, and PRF1 and the downregulation of CD28 (Fig. 2c) [22].

We assessed the RNA levels of transcription factors known to be upregulated in exhausted T cells [42] and detected high expression of IKZF2, EGR2, and ZEB2 and a trend of TOX and IRF4 in SLAMF7^+^CD8^+^ T cells compared with SLAMF7^−^CD8^+^ T cells (Fig. 2d), further indicating the exhausted state of these cells.

The gene set enrichment analyses (GSEAs) revealed significant upregulation of various pathways [43], including “positive regulation of interleukin-6 production” (*p* = 0.001); IL-6 drives myeloma cell survival and its secretion can be inhibited by lenalidomide therapy [44]. In addition, “reactome immunoregulatory interactions between a lymphoid and a nonlymphoid cell” were upregulated in SLAMF7^+^ T cells (*p* = 0.001); this pathway regulates the response of T cells to self and tumor antigens (Fig. 2e).

### SLAMF7^+^CD8^+^ T cells impairs antigen-specific T cell responses

**Fig. 3:**
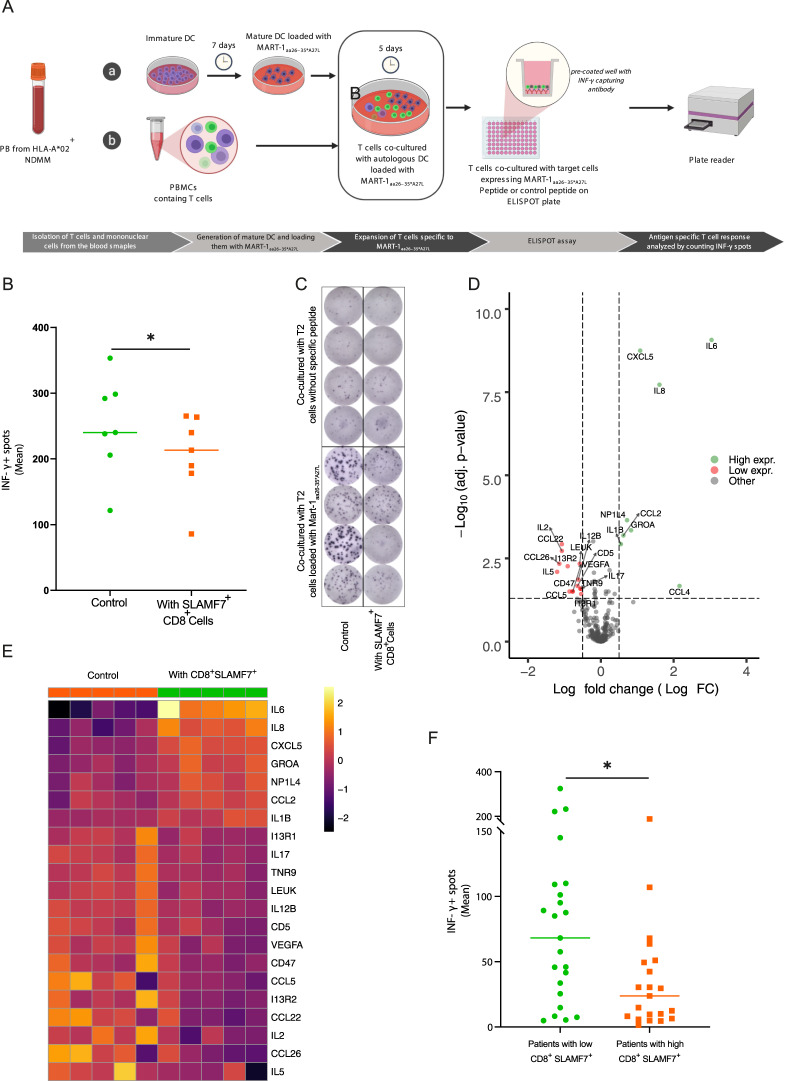
SLAMF7 + CD8 + T cells impairs antigen-specific T cell responses. **a** Schematic figure describing the MART-1_aa26–35*A27L_ ELISPOT antigen-specific T cell model. **b** Scatter plots showing the effect of adding CD8^+^SLAMF7^+^ from the BM of NDMM during the expansion of HD antigen-specific T cells (*n* = 7). **c** Microscopic photo highlights the difference in the frequency of IFN-γ spots between CD8^+^ T cells cultured with (right) or without (left) SLAMF7^+^CD8^+^ cells in the ELISPOT wells. **d** Volcano plot showing the differential expressed proteins in the supernatants of T cell cultures. Green dots represent proteins differentially expressed in the CD8^+^SLAMF7^+^ containing cell cultures, red dots represent proteins differentially expressed in the control cell cultures (*n* = 5). **e** Heatmap showing the top differentially expressed proteins. **f** Scatter plots showing the effect of CD8^+^SLAMF7^+^ abundance on antigen-specific T cell response (measured by the mean of IFN-γ spots) in 45 NDMM patients (*p* = *0.01*). Differences between groups were compared using Mann Whitney test; ^∗^*p* < 0.05, ^∗∗^*p* < 0.01, and ^∗∗∗^*p* < 0.001.

We used the myeloma antigen MART-1_aa26–35*A27L_ ELISPOT model [45] to analyze the suppressive capacity of SLAMF7^+^ CD8^+^ T cells on antigen-specific response. In this model T cells specific to MART-1_aa26–35*A27L_ peptide were expanded by co-culturing them with autologous dendritic cells pre-loaded with the MART-1_aa26–35*A27L_ peptide for 5 days. (Fig. 3a). During the expansion, SLAMF7^+^ CD8^+^ T cells isolated from the BM of NDMM were added in a trans-well plate, that allowed the exchange of cytokines by a pored membrane without direct cell-cell interaction. Healthy CD8 + T cells expanded in the existence of SLAMF7^+^ CD8^+^ cells from NDMM showed significantly weaker cytotoxic function against target cells in comparison to the control cells (Fig. 3b, c).

High throughput screening of 351 different cytokines and immune proteins in the supernatants of the T cells cultures revealed strong upregulation of IL-6, IL-8, both vital survival factors for myeloma cells [46–48], and CXCL5, a chemokine that could enhance the frequency of CD4 Treg [49], in the cultures containing CD8^+^SLAMF7^+^ cells. IL-2 and IL-5 were upregulated in the control cultures without CD8^+^SLAMF7^+^ T cells, highlighting a more activated state in the control group and suggestion IL-6 and IL-8 as potential effector cytokines for the suppressive CD8^+^SLAMF7^+^ T cells (Fig. 3d, e, full list of all analyzed protein in Data Table S2).

We then used the same MART-1_aa26–35*A27L_ ELISPOT model to analyze the effect of CD8^+^SLAMF7^+^ T cells abundance on antigen-specific T cell responses in 45 NDMM patients. Patients with high SLAMF7^+^CD8^+^ T cells (higher than or equal to the median SLAMF7^+^CD8^+^ frequency of all patients) showed significantly lower antigen-specific T-cell response in comparison to patients with low SLAMF7^+^CD8^+^ T cells (lower than the median SLAMF7^+^CD8^+^ frequency of all patients), (Fig. 3f).

### Effect of elotuzumab induction therapy on SLAMF7^+^CD8^+^ Tregs in patients with symptomatic MM

**Fig. 4:**
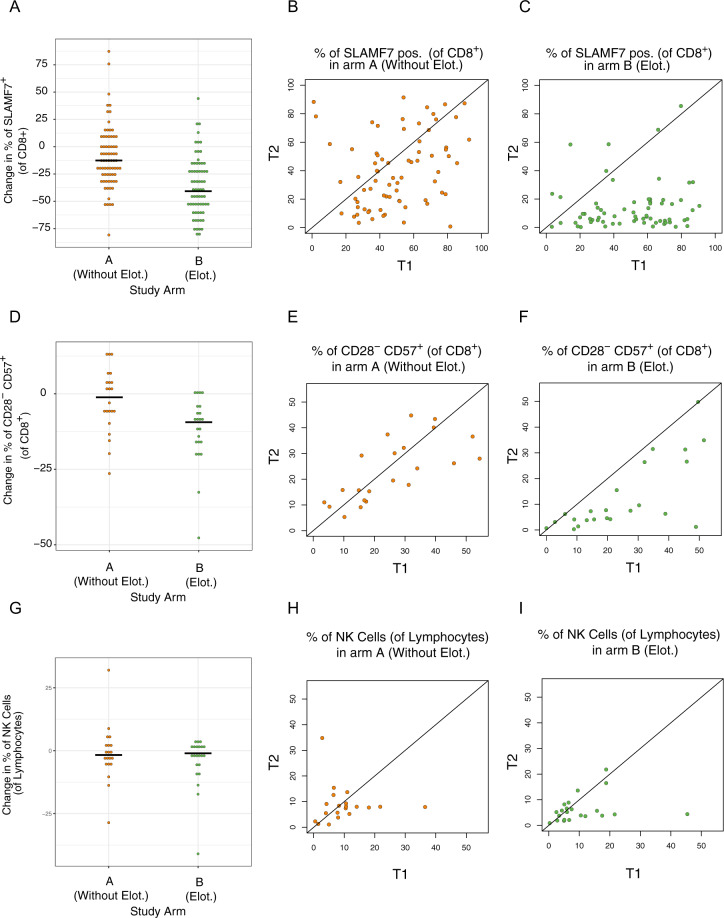
Effect of elotuzumab induction therapy on SLAMF7^+^CD8^+^ Tregs in patients with symptomatic MM. **a**–**c** Scatter plots showing the difference in the frequencies of CD8^+^ T cells expressing SLAMF7 (of total CD8^+^ cells) before (T1) and after (T2) induction therapy, with each dot representing one patient (*n* = 73 in study arm A, *n* = 67 in study arm B). **d**–**f** Scatter plots showing the difference in the percentage of CD8^+^ Treg cells (of total CD8^+^ cells) before (T1) and after (T2) induction therapy, with each dot representing one patient (*n* = 22 in study arm A, *n* = 23 in study arm B). **g**–**i** Scatter plots showing the difference in NK cell percentages (of total lymphocytes) before (T1) and after (T2) induction therapy, with each dot representing one patient (*n* = 20 in study arm A, n = 22 in study arm B). Differences between groups were evaluated using Student’s *t*-test; ^∗^*p* < 0.05, ^∗∗^*p* < 0.01, and ^∗∗∗^*p* < 0.001.

We then analyzed the effects of anti-SLAMF7 antibody (elotuzumab) on SLAMF7 expressing T cells. Patients with NDMM were treated within the GMMG HD6 trial (NCT02495922), in which patients were randomized into two study arms for induction therapy. Patients in study arm A received 4 cycles of bortezomib, lenalidomide, and dexamethasone (VRD) as an induction therapy. Patients in study arm B received 4 cycles of VRD and elotuzumab (10 mg/kg on days 1, 8, and 15 in the induction cycles 1 and 2 and on days 1 and 11 in the induction cycles 3 and 4) as induction therapy [23]. Data were analyzed from 265 patients before and after induction therapy (Table 1 and Supplementary Table S1). Addition of elotuzumab was associated with a loss of SLAMF7^+^ CD8^+^ T cells after the induction therapy (median frequencies before and after induction therapy of 49.4% and 7.7% respectively, *p* < 0.0001). Of note, a slight reduction in the CD8^+^ SLAMF7^+^ cell frequency was also observed in patients in study arm A (median frequencies before and after induction therapy of 44.9–35.6% respectively, *p* = 0.0007). These findings suggested a direct effect of elotuzumab on SLAMF7^+^ CD8^+^ cells (Fig. 4a–c).

**Table 1:** Clinical characteristics and prognostic factors.

Characteristics		A (Without Elotuzumab)		B (Elotuzumab)		All	
Age	Median (range)	59 (41–70); 1 missing		59 (33–70)		59 (33–70); 1	
		*n*	(%)	*n*	(%)	*n*	(%)
Sex *p* = 0.81	Female	66	47.5	57	45.2	123	**46.4**
	Male	73	52.5	69	54.8	142	**53.6**
Heavy chain type *p* = 0.66	IgA	23	16.6	22	17.5	45	**17.0**
	IgG	90	64.8	79	62.7	169	**63.8**
	Other (IgM, IgD, IgE)	0	0.0	2	1.6	2	**0.8**
	None	26	18.7	23	18.2	49	**18.5**
Light chain type *p* = 0.70	Kappa	91	65.5	79	62.7	170	**64.2**
	Lambda	48	34.5	47	37.3	95	**35.9**
ISS^a^ *p* = 0.52	I	56	40.3	59	46.8	115	**43.4**
	II	49	35.2	42	33.3	91	**34.3**
	III	34	24.5	25	19.8	59	**22.3**
Cytogenetic risk group^b^ *p* = 0.88	0	73	65.2	67	67.0	140	**66.0**
	1	39	34.8	33	33.0	72	**34.0**
	**missing**	**27**		**26**		**53**	

The bold values are the percentage of each subgroup from total patients.

^a^ISS International Staging System.

^b^Cytogenetic high-risk group: presence of del17p and/or *t*(4;14) or *t*(14;16).

A similar effect was observed when comparing CD8^+^ Treg frequencies between the study arms in 45 patients before and after the induction therapy. CD8^+^ Treg cells decreased in study arm B (*p* < 0.0001) whereas no effect was observed in study arm A (*p* = 0.41, Fig. 4d–f). Among NK cells, which also express SLAMF7, we observed a slight but not significant decrease in the percentages of NK cells among total lymphocytes in patients in study arm B (*p* = 0.08), no change was observed in patients in study arm A (*p* = 0.35, Fig. 4g–i).

We analyzed EAT-2 expression to investigate whether the decrease of SLAMF7 expressing CD8^+^ cells occurred due to elotuzumab-induced activation of the SLAMF7 downstream pathway. The flow cytometry analyses revealed that SLAMF7^+^CD8^+^ T cells expressed high EAT-2 protein levels (Supplementary Fig. S2A). EAT-2 binds to SLAMF7-L via phosphorylated tyrosine 281 (Y281) and thereby triggers a downstream pathway that eventually activates phospholipase C-γ (PLC-γ) in NK cells in mice [50, 51]. We confirmed the expression of both SLAMF7 isoforms in CD8^+^ T cells (data not shown). We hypothesized that elotuzumab could activate the downstream pathway in SLAMF7^+^CD8^+^ T cells. But, cytokine secretion by CD8^+^ T cells was not affected by incubation with 100 µg/ml elotuzumab for 48 h except for a slight decrease in interferon gamma (IFN-γ) secretion in the elotuzumab-treated samples (Supplementary Fig. S2B). Furthermore, the Annexin V/PI analysis excluded elotuzumab-induced ADCC of CD8^+^ T cells (Supplementary Fig. S2C).

### Elotuzumab induced the antibody-mediated phagocytosis of SLAMF7^+^CD8^+^ T cells

**Fig. 5:**
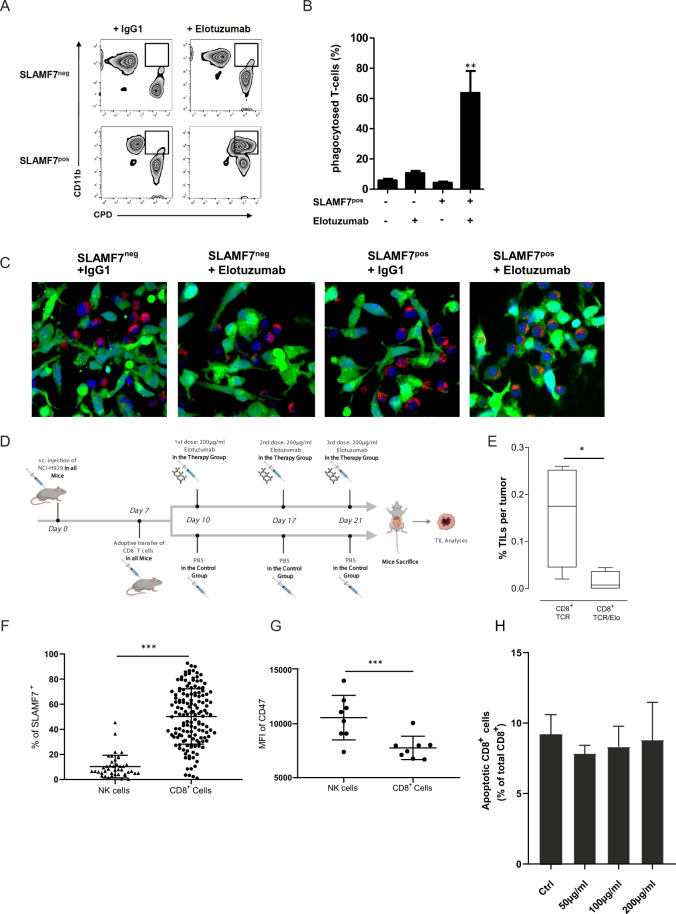
Elotuzumab induces antibody-mediated phagocytosis of SLAMF7^+^CD8^+^ T cells. **a** Macrophages were co-incubated with autologous CPD-labeled T cells (E:T = 1:1) in the presence or absence of elotuzumab (10 μg/ml) or control IgG1 antibody for 24 h. To distinguish between phagocytosed CPD-positive T cells and free T cells, macrophages were counterstained with an anti-CD11b antibody and analyzed by flow cytometry. **b** Bar graph showing the mean percentage of phagocytosed T cells (CD11b^+^ and CPD^+^) from six independent experiments. **c** CPD-labeled T cells (red) were added to autologous macrophages (stained with CFSE, green) as effectors at an E/T ratio of 1:1 in the presence or absence of elotuzumab or control IgG1 antibody (10 μg/ml). Samples were counterstained with DAPI (blue). After 2 h, phagocytosis was analyzed by confocal microscopy at 630X. Scale bar: 10 μm. **d** Representative figure describing the mouse model experiment. **e** Bar graph showing the mean percentage of CD8^+^ T cells per tumor with or without elotuzumab therapy from 4 different mice (*p* = 0.043). **f** Scatter plot showing the percentage of NK (from total lymphocytes) and CD8^+^ T cells expressing SLAMF7 (from total CD8^+^ compartment) from the PB of NDMM patients (*n* = 42 and *n* = 146, respectively); each dot represents a single patient (*p* < 0.0001). **g** Scatter plot showing the mean fluorescence intensity (MFI) of CD47 for both NK and CD8^+^ T cells from the PB of NDMM patients (*n* = 8, *p* < 0.001). **h** Bar graph showing the mean frequency of apoptotic CD8 + T cells (% of total CD8^+^) in varying concentrations of elotuzumab (*n* = 4). Differences between groups were evaluated using Student’s *t*-test; ^∗^*p* < 0.05, ^∗∗^*p* < 0.01, and ^∗∗∗^*p* < 0.001.

To further explore the mechanism of depletion of SLAMF7^+^ T cells in patients who received elotuzumab induction therapy, we analyzed the potential role of macrophages in this process. Macrophages generated from HDs were coincubated with sorted autologous SLAMF7^+^ or SLAMF7^−^ T cells labeled with Cell Proliferation Dye (CPD) at an effector:target ratio (E:T) of 1:1 in the presence or absence of elotuzumab (10 µg/ml) or control IgG1 antibody for 24 h. To distinguish between phagocytosed CPD-positive T cells and free T cells, macrophages were counterstained with an anti-CD11b antibody and analyzed by flow cytometry and confocal microscopy. Interestingly, we found that elotuzumab caused strong antibody-dependent cellular phagocytosis (ADCP) of SLAMF7^+^ T cells but not SLAMF7^−^ T cells, with no observed effect of control IgG1 antibody (Fig. 5a–c), suggesting that direct macrophage-mediated ADCP of SLAMF7^+^ T cells is a mechanism of elotuzumab.

We then further addressed the potential depletion of SLAMF7^+^CD8^+^ T cells by elotuzumab in vivo using a mouse T cell transfer model. To test this hypothesis, NOD/SCID/IL2rγ^null^ (NSG) mice were injected subcutaneously (s.c.) with NCI-H929 myeloma cells and then received an adoptive intravenous (i.v.) transfer of tumor-associated antigen T cell receptor (TCR)-redirected human CD8^+^ T cells, highly expressing SLAMF7, 7 days later. Mice were further divided into two groups that received either elotuzumab or PBS. After treatment, the mice were sacrificed, and tumor-infiltrating T cells (TILs) were isolated from freshly extracted tumors and analyzed by flow cytometry (Fig. 5d). The results showed a reduction in CD8^+^ TIL density in mice treated with elotuzumab compared with PBS-treated mice (Fig. 5e).

Having confirmed that the observed depletion of SLAMF7^+^CD8^+^ T cells in vitro is due to ADCP, we then sought to explore the factors hindering a similar effect on NK cells despite their SLAMF7 expression. We hypothesized that ADCP depends on the proportion of cells expressing the marker, so we examined the differences in the proportions of CD8^+^ T cells and NK cells expressing SLAMF7. We found that a significantly higher proportion of CD8^+^ T cells versus NK cells expressed SLAMF7 (Fig. 5f). Moreover, we compared the expression level of CD47, a phagocytosis inhibitory (“don’t eat me”) marker, on both CD8^+^ T cells and NK cells; strikingly, NK cells expressed higher levels of CD47 than T cells (Fig. 5g).

To examine whether NK cells could contribute to the depletion of SLAMF7^+^ CD8^+^ T cells by inducing an ADCC process during elotuzumab therapy, we co-cultured T cells with high SLAMF7 expression level with autologous NK cells at varying concentrations of elotuzumab (50, 100, or 200 µg/ml) and then tested the cells for apoptosis using flow cytometry. No significant difference was observed in the frequency of apoptotic CD8^+^ T cells between the different samples, suggesting that NK cells are unlikely to induce ADCC in T cells (Fig. 5h).

## Discussion

In this study, we demonstrate the capacity of antibody-based therapy to eliminate a subpopulation of CD8^+^ T cells expressing SLAMF7 using in vivo data from MM patients as well as in vitro assays and an in vivo murine model. Most CD8^+^ Tregs expressed SLAMF7 and coexpressed exhaustion markers, including PD-1 and TIGIT. Furthermore, SLAMF7^+^CD8^+^ T cells frequency in the PB of NDMM patients was correlated with impaired antigen-specific T cell responses. While single-agent therapy targeting PD-1/PD-L1 was not promising in MM [52, 53], recent data provide strong evidence that TIGIT might be a candidate for immunotherapy in MM. TIGIT was found to induce myeloma immune escape after transplantation, and blocking TIGIT could overcome tumor progression [41]. The current study provides evidence that exhausted cytotoxic T cells which express TIGIT and share the Treg phenotype can be depleted by ADCP using anti-SLAMF7 antibody.

Data from the GMMG HD6 trial (NCT02495922), in which patients with NDMM were randomized for induction therapy with 4 cycles of either VRD only or VRD and elotuzumab, showed no clinical correlation of SLAMF7 expression on T cells with the response after induction (data not shown). This likely reflects the challenge in detecting such a significant correlation during highly efficient first line therapy of MM, which results in remission for most patients.

Notably, a recent study showed that SLAMF7 expression on target cells and binding to SLAMF7 on phagocytes mediated by coupling to Mac-1 are critical for the elimination of tumor cells by phagocytosis in CD47-blocking therapy [54]. In contrast, another report showed that SLAMF7 coexpression on macrophages and tumor cells is not required for CD47-mediated phagocytosis [55]. As SLAMF7 expression in macrophages was not determined in this clinical study, we cannot conclude whether SLAMF7 expression on this ADCP-mediating cell type plays a role in enhancing the elotuzumab-mediated elimination of SLAMF7^+^CD8^+^ T cells in MM patients.

In summary, we have identified antibody-mediated phagocytosis of CD8^+^ Tregs as a possible novel immunomodulatory effect of elotuzumab, highlighting a role for the elimination of these Tregs in clinical outcomes. Moreover, it is conceivable that elotuzumab could benefit patients with other tumors characterized by the upregulation of CD8^+^ Tregs [56–58].

## Supplementary information

Supplementary Materials and Figures

Table S1

Data Table S1

Data Table S2
