# Supplementary material for: Selective elimination of immunosuppressive T cells in patients with multiple myeloma
Source: Leukemia. 2021 Feb 17;35(9):2602–15. doi: 10.1038/s41375-021-01172-x (PMC8410603; doi:10.1038/s41375-021-01172-x)
Supplement: Supplementary file 1 — Supplementary Materials and Figures [file 41375_2021_1172_MOESM1_ESM.pdf]

Supplementary Materials and Figures

Supplementary Methods
Suppl. Figure S1: SLAMF7 expression and Knock down effect.
Suppl. Figure S2: Effect of elotuzumab on SLAMF7 expressing cells.

Supplementary Methods

Cell culture medium and reagents

Unless otherwise specified, T cells were maintained in T cell medium comprising RPMI 1640 cell culture medium, penicillin/streptomycin, 2 mM L-glutamine (all from PAA Laboratories, Pasching, Austria), 5% heat-inactivated human serum (PAA Laboratories) and 50 IU/ml IL-2 (Proleukin, Chiron GmbH, Munich, Germany). Unless otherwise indicated, reagents were purchased from Sigma (Sigma-Aldrich, Steinheim, Germany).

Isolation of mononuclear cells (MNCs)

MNCs were isolated from PB or BM using Ficoll-Hypaque density gradient centrifugation (Biochrom, Berlin, Germany).

Flow cytometry

The expression of T cell surface markers was analyzed by flow cytometry. Cells were suspended in PBS and incubated according to the manufacturer's instructions with the following fluorochrome-labeled antibodies:

- APC-H7 mouse anti-human CD45 clone 2D1 (#560178).
- PE-Cy7 mouse anti-human CD3 clone SK7 (#557851).
- PerCP mouse anti-human CD8 clone SK1 (#347314).
- PE mouse anti-human CD28 clone CD28.2 (#555729).
- Alexa Fluor 647 mouse anti-human CRACC (CD319 or SLAMF7) clone 235614 (#564338).
- PE mouse anti-human CD197 (CCR7) clone 150503 (#560765).
- V450 mouse anti-human CD45RA clone HI100 (#560362).
- PE mouse anti-human CD152 (CTLA-4) clone BNI3 (#557301).
- PE-Cy7 mouse anti-human CD62L (#565535).
- BV421 mouse anti-human CD279 (PD-1) clone EH12.1 (#562516).
- PE mouse anti-human CD16 clone 3G8 (#555407).
- BV421 mouse anti-human CD57 clone NK-1 (#563896).
- V450 mouse anti-human CD56 clone B159 (#560360).
- PE mouse anti-human LAG-3 (CD223) clone T47-530 (#565616).
- BV421 mouse anti-human TIM-3 (CD366) clone 7D3 (#565562).

  (from BD Biosciences, Heidelberg, Germany)

- PE/Cy7 mouse anti-human TIGIT clone A15153G (#372714).
- FITC anti-human CD47 clone CC2C6 (#323106)

  (From BioLegend, Koblenz, Germany).

Control cells were stained with the corresponding isotype antibodies at the same concentration. Flow cytometry analyses were performed on a BD FACSLYRIC flow cytometer with BD FACSDiva software; data were analyzed using FlowJo software.

CRISPR-Cas9 SLAMF7 knockout

To analyze the functional role of SLAMF7 expression in $CD8^+$ T cells, Permanent genome editing of healthy human $CD8^+$ T cells was achieved by the CRISPR-Cas9 RNP and synthetic guide RNA (sgRNA) electroporation approach using Cas9-GFP from Sigma-Aldrich (Steinheim, Germany). Briefly, $CD8^+$ T cells were enriched using a MACS kit (Miltenyi Biotec, Bergisch Gladbach, Germany) according to the manufacturer's protocol. The cells were then activated using Human CD3/CD28/CD2 T Cell Activator and cultured in ImmunoCult™-XF T Cell Expansion Medium (both from STEMCELL Technologies, Köln, Germany). After 2 days, the cells were electroporated with Cas9-GFP preincubated with either sgRNA targeting SLAMF7 (crRNA sequence: 5' AAA GAG CUG GUC GGU UCC GU 3') or Scrambled sgRNA (crRNA sequence: 5' GUA UUA CUG AUA UUG GUG GG 3') using the Invitrogen Neon Transfection System (Thermo Fisher, Germany). The electroporation parameters were adjusted to 1400 V, pulse length of 30 ms and 1 pulse. The cells were then directly cultured in prewarmed ImmunoCult™-XF T Cell Expansion Medium for 2 h, after which the medium was changed, and the The cells were cultured for 2 more days. and Knockdown efficiency was then assessed using flow cytometry analysis.

Expansion of MART-1$_{aa26\text{-}35*A27L}$-specific T-cells and IFN-γ ELISPOT assay

T-cells specific for the antigen MART-1$_{aa26\text{-}35*A27L}$ show cross-reactivity for HM1.24, a highly-expressed antigen on MM cells, and are able to lyse autologous MM cells. PBMCs from "HLA-A*02$^+$" HD were used to generate MART-1aa26-35*A27L-specific T-cells using autologous pre-loaded dendritic cells as previously described[24]. $CD8^+$ cells purified from the MART-1$_{aa26\text{-}35*A27L}$-activated T-cell population were then incubated with the MART-1$_{aa26\text{-}35*A27L}$ peptide or irrelevant peptide-pulsed T2 cells and the killing was assessed using INF-γ ELISPOT assay as previously described[24]. Immature DCs were obtained by culturing plastic-adherent PBMCs for 5 days in RPMI

1640 medium containing GM-CSF (800 U/ml, Sargramostim, Bayer, Seattle, WA, USA), IL-4 (500 U/ml, R&D Systems, Minneapolis, MN, USA) and 5% heat-inactivated human serum. The maturation of immature DCs was then induced by supplementing TNF-α (10 ng/ml, R&D Systems), IL-6 (1000 U/ml, PromoCell) and prostaglandin E_2 (1 µg/ml, Biomol/Enzo Lifesciences, Lörrach, Germany) for 2 days in the presence of the MART-$1_{aa26\text{-}35*A27L}$ peptide (10 µg/ml) to load the DCs. Afterwards, autologous PBMCs were incubated for 7 days together with mature DCs loaded with MART-$1_{aa26\text{-}35*A27L}$ peptide in T-cell medium to expand the MART-$1_{aa26\text{-}35*A27L}$-specific T-cells. $CD8^+$ cells were purified from the MART-$1_{aa26\text{-}35*A27L}$-activated T-cell population by positive immunomagnetic cell sorting (MACS-system, Miltenyi Biotec). Purified $CD8^+$ cells were then incubated with the MART-$1_{aa26\text{-}35*A27L}$ peptide or irrelevant peptide-pulsed T2 cells (loaded by a 2-h incubation in serum-free RPMI 1640 media containing 10 µg/ml peptide) for 24 h in anti-IFN-γ antibody- (Mabtech, Nacka Strand, Sweden) coated nitrocellulose-plates (Millipore, Schwalbach, Germany) in an effector cell to target cell (E:T) ratio of 1:5. Subsequently, plate-bound IFN-γ was detected according to manufacturer instructions. ELISPOT experiment was considered functional if at least 10 dots were detected and if the mean of the mean of the MART-$1_{aa26\text{-}35*A27L}$ wells showed more dots than the control peptide wells.

SLAMF7 isoform PCR

RNA was isolated from dry cell pellets using TRIzol method. cDNA was synthesized from RNA using a High-capacity cDNA Reverse Transcription Kit (Thermo Fisher) according to the manufacturer´s instructions. The following primers were used to amplify SLAMF7: forward 5`GTG ACC AAT CTG ACA TGC TGC 3´ and reverse 5´CTG CTC ACG ATG CCA GAC AC 3´. PCR was performed using AmpliTaq Gold DNA polymerase (Thermo Fisher) with 0.2 µM of each primer and 0.2 mM of each dNTP. The PCR program was as follows: initial denaturation at 95°C for 5 min, followed by 40 cycles of denaturation (95°C, 30 s), annealing (64°C, 1 min), and elongation (72°C, 1 min), with a final elongation step at 72°C for 5 min.

Nonspecific activation of T cells and enzyme-linked immunosorbent assay (ELISA)

To analyze the effect of elotuzumab on the nonspecific activation of T cells, $CD3^+$ T cells were isolated from MNCs using the MACS System and were activated with anti-CD3/CD28 microbeads (Dynabeads, Invitrogen Dynal, Oslo, Norway) for 24 h at a

cell:bead ratio of 1:4. Afterwards, the concentrations of granzyme B, IFNγ, IL-2 and perforin in the culture supernatants were determined with ELISA kits (Mabtech, Germany) according to the manufacturer's instructions. Briefly, ELISA microplates (Greiner Bio-One, Frickenhausen, Germany) were coated with a capture antibody overnight, washed twice with PBS and blocked for 1 h with blocking buffer (PBS with 0.05% Tween 20 and 0.1% BSA); the plates were then washed 5 times with wash buffer (PBS containing 0.05% Tween 20). Cell supernatants or standards were then added to the wells, and the plates were incubated for 2 h at room temperature. Then, the plates were washed, and a detection antibody was added for 1 h. A streptavidin solution was added to the wells for 1 h. After a final wash, an appropriate substrate solution was added, and the optical density was measured with an ELISA reader.

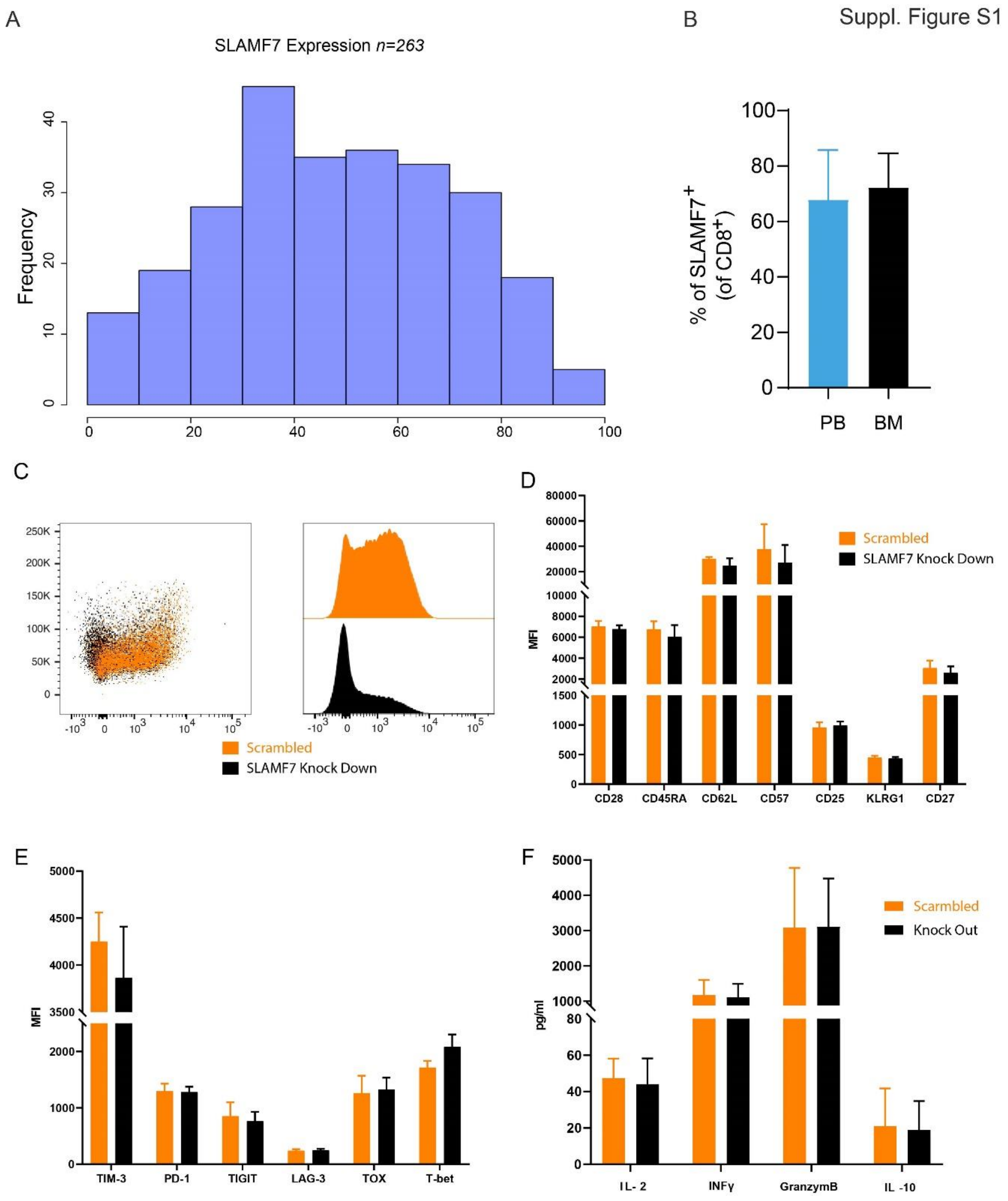

Supplementary Figure S1

(A) Histogram graph showing the frequency of SLAMF7 expressing $CD8^+$ cells in 263 MM patients.

(B) Bar graph showing the differences between the percentages of $CD8^+$ cells expressing SLAMF7 in the PB and BM from identical NDMM patients (n=5).

(C) Representative flow cytometry dot plot (left) and histogram plot (right) showing the difference in SLAMF7 expression on $CD8^+$ T cells in scrambled (in orange) and knockout (in black) groups.

(D) Bar graph showing the effects of SLAMF7 knockout on T cell differentiation markers (n=4).

(E) Bar graphs showing the effects of SLAMF7 knockout on the expression of T cell exhaustion markers (n=4).

(F) Bar graphs showing the effects of SLAMF7 knockout on the secretion of different cytokines (n=5).

Differences between groups were evaluated using Student's t-test; $*p < 0.05$, $**p < 0.01$, and $***p < 0.001$.

Suppl. Figure S2

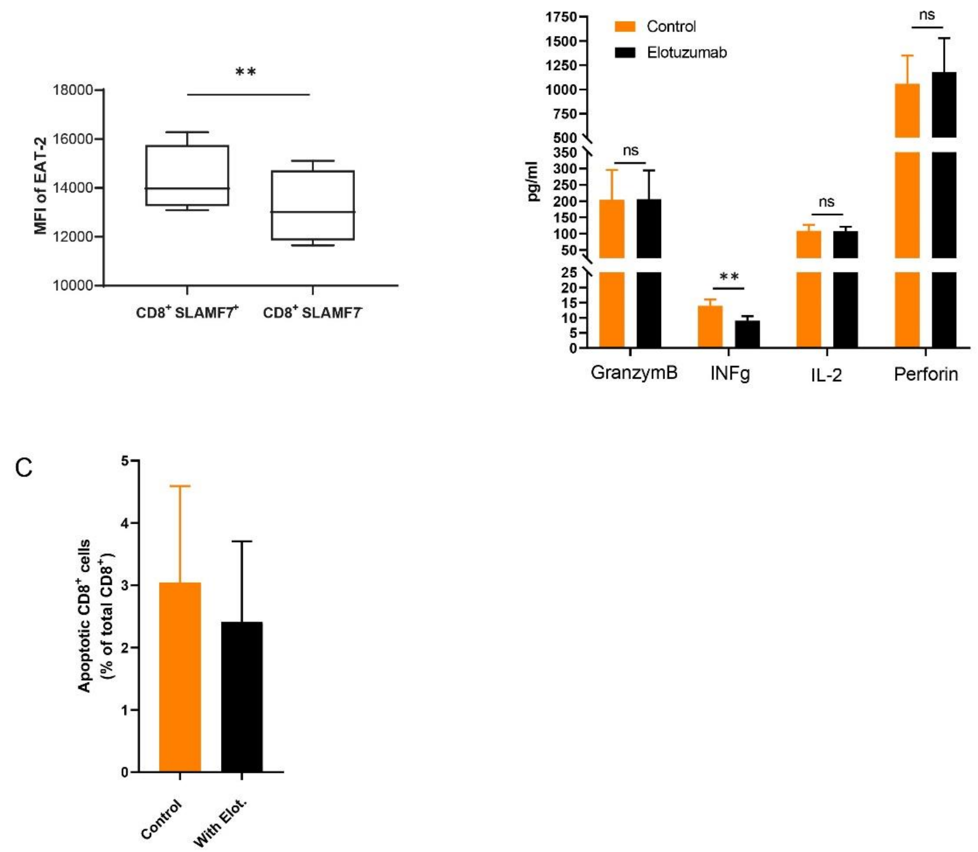

Supplementary Figure S2

(A) Box plot showing the difference in EAT-2 mean fluorescence intensity (MFI) between SLAMF7^{+} and SLAMF7^{-} CD8^{+} cells (n=4).

(B) Bar graph showing the effect of elotuzumab on the secretion of cytokines (in pg/ml) from CD8^{+} cells isolated from the BM of MM patients (n=4).

(C) Bar graph showing the different in the percentage of apoptotic CD8^{+} cells from total CD8^{+} cells between the control (orange) and elotuzumab (black) (n=4).
